# Computational Identification of GABA-A Receptor Ligands Targeting the Histamine Allosteric Pocket

**DOI:** 10.3390/cimb48101078

**Published:** 2026-10-19

**Authors:** Dragos Paul Mihai, Corina Cornet, George Nicolae Daniel Ion, Georgiana Nitulescu, Octavian Tudorel Olaru, Anca Zanfirescu, Mehtap Kara, Ezgi Öztaş, George Mihai Nitulescu

**Affiliations:** 1 Faculty of Pharmacy, “Carol Davila” University of Medicine and Pharmacy, Traian Vuia 6, 020956 Bucharest, Romania; dragos_mihai@umfcd.ro (D.P.M.); george-nicolae-daniel.ion@umfcd.ro (G.N.D.I.); georgiana.nitulescu@umfcd.ro (G.N.); octavian.olaru@umfcd.ro (O.T.O.); anca.zanfirescu@umfcd.ro (A.Z.); george.nitulescu@umfcd.ro (G.M.N.); 2 Department of Pharmaceutical Toxicology, Faculty of Pharmacy, Istanbul University, Istanbul 34452, Türkiye; mehtap.kara@istanbul.edu.tr (M.K.); ezgi.oztas@istanbul.edu.tr (E.Ö.)

**Keywords:** GABA-A receptor, histamine allosteric pocket, allosteric ligands, ligand-based similarity, molecular docking, molecular dynamics, ADMET prediction

## Abstract

The γ-aminobutyric acid type A (GABA-A) receptor is a key regulator of inhibitory neurotransmission in the central nervous system, and a promising target for cognitive and neurological disorders. Histamine interacts with a distinct allosteric pocket located at the β3–β3 subunit interface, providing an opportunity for the discovery of novel ligands. In the present study, an integrated in silico workflow combining ligand-based similarity analysis, ADMET filtering, molecular docking, and molecular dynamics simulations was applied to identify candidate ligands for this histamine-associated site. Docking analysis showed that ligand efficiency-related metrics discriminated active from inactive ligands more effectively than docking score alone, supporting their use during virtual screening. Three compounds were evaluated by 100 ns molecular dynamics simulations, which showed preserved receptor structural integrity and stable protein–ligand complexes. CHEMBL3236582 exhibited the most favorable dynamic profile, combining low backbone and ligand RMSD (root mean square deviation) values with the most favorable Molecular Mechanics/Poisson–Boltzmann Surface Area (MM/PBSA) binding energy estimate, whereas CHEMBL521996 maintained stable binding despite adopting an alternative binding orientation. Overall, the integrated computational workflow identified CHEMBL3236582 as the most promising candidate for experimental validation and supports further investigation of the histamine-associated allosteric pocket for the discovery of novel GABA-A receptor ligands.

## 1. Introduction

GABA-A receptors are key mediators of inhibitory neurotransmission in the central nervous system (CNS), where they help maintain the physiological balance between neuronal excitation and inhibition [1]. Functionally, they form chloride-permeable channels that open following γ-aminobutyric acid (GABA) binding at the α–β subunit interface, triggering conformational changes that allow chloride influx, membrane hyperpolarization, and suppression of neuronal excitability [2,3]. Structurally, GABA-A receptors are heteropentameric transmembrane complexes assembled from multiple subunit families, including α1–6, β1–3, γ1–3, δ, ε, π, and θ. The predominant brain configuration typically comprises two α, two β, and one γ subunit, and α1-containing receptors represent the most abundant subtype in the brain and are associated with sedative effects; however, alternative stoichiometries have also been reported (e.g., assemblies containing three β3 subunits) [4]. Because impaired GABAergic signaling is implicated in several CNS disorders, such as epilepsy, anxiety, insomnia, and cognitive deficits, these receptors represent important targets for therapeutic modulation [5].

Notably, GABA-A receptor α4 and α5 subunits show distinct regional expression and functional contributions. The α4 subunit is most prominent in the thalamus, where it helps regulate relay neuron activity involved in sensory processing and sleep, whereas α5 subunits are enriched in the hippocampus and are linked to cognitive processes by supporting network plasticity underlying learning and memory [6,7]. Both subtypes are implicated in tonic inhibition, which is commonly mediated by extrasynaptic, δ-containing GABA-A receptors that remain active in response to ambient extracellular GABA, thereby constraining neuronal excitability [8,9].

Pharmacological regulation of GABA-A receptors includes both positive and negative allosteric modulation. Positive allosteric modulators (PAMs), including benzodiazepines, barbiturates, neurosteroids, and anesthetics, enhance inhibition, while negative allosteric modulators (NAMs), especially those targeting α5 receptors, are being explored to support cognition with a lower risk of sedation [10,11]. Additionally, gaboxadol, an agonist selective for extrasynaptic δ-containing receptors, enhances tonic inhibition by increasing chloride conductance, but failed as an insomnia hypnotic due to adverse effects, variable efficacy, tolerance, and withdrawal [12,13,14].

In addition to its wake-promoting effects mediated by histamine G protein–coupled receptors, histamine can also directly potentiate GABA-A receptor function as a positive allosteric modulator. This effect appears to preferentially enhance α4-containing receptors and, to a lesser extent, α5-containing receptors, suggesting a link between histaminergic signaling and sleep–wake regulation, including sedation when extrasynaptic receptors are engaged [6,15,16]. This action is mediated by a distinct allosteric pocket in β3-containing receptor assemblies, where histamine binds at the β3–β3 subunit interface rather than at the GABA or benzodiazepine sites [17]. By contrast, several histamine analogues exhibit inverse agonist effects and can variably inhibit GABA-A receptor currents via allosteric mechanisms in transfected cells [17,18].

In pathological contexts, such as stroke, excessive tonic inhibition mediated by α5-containing receptors may limit plasticity and impair recovery, whereas α5-selective NAMs (e.g., L-655,708) have been linked to improved cognitive recovery without triggering seizures [19]. In parallel, stroke, cognitive disorders, and neurodegenerative dementias share overlapping mechanisms, including oxidative stress, mitochondrial dysfunction, and apoptosis, that contribute to neuronal loss and cognitive decline [20,21,22].

Building on this framework, the present study aimed to identify candidate ligands targeting the histamine-associated allosteric pocket of the GABA-A receptor. The novelty of the present study lies in the focused exploration of the β3–β3 histamine-associated pocket using experimentally characterized histaminergic ligands as reference compounds and in the integration of ligand-based similarity analysis, predicted ADMET properties, docking-derived efficiency metrics, and molecular dynamics simulations into a unified prioritization workflow. This strategy was designed to generate experimentally testable candidate ligands rather than to establish their pharmacological activity in the absence of functional validation.

## 2. Materials and Methods

### 2.1. Dataset Preparation

A virtual ligand library was assembled and used for structure–activity relationship (SAR) analyses and further docking and ADMET screening (evaluation set E). The library was constructed starting from the chemical structures of histaminergic receptor modulators (H1–H4), retrieved from the ChEMBL database. The collected data underwent a standard database cleaning and standardization workflow (covering more than 8000 molecules), which included duplicate removal, structural consistency checks, and exclusion of incomplete or ambiguous entries. Salts, solvents, and inappropriate molecular fragments were removed, and the chemical structures were standardized with respect to their representation. A reference library of known active binders (set A, *n* = 16) and inactive molecules (set I, *n* = 35) was retrieved from a previously published study that evaluated the activity of 51 histaminergic ligands against GABA-A receptors in homopentameric β3 configuration [23].

### 2.2. Structure Analysis

A dataset of 5641 unique molecules, comprising the evaluation set (set E), was evaluated for similarity against the 16 active reference molecules in set A. Similarity calculations were performed in DataWarrior v06.05.02 [24] using a threshold of 0.85 across multiple descriptor types: FragFp, PathFp, AllFragFp, SphereFp, SkelSpheres, OrgFunctions, and Flexophore (detailed in Table 1). For each molecule in set E, a global activity score (GA) was computed by summing the number of active reference molecules to which it was similar across all fingerprints.

The Descriptor Consensus Score (DCS) was calculated as an additive binary measure across descriptor types. For a given molecule, a descriptor was counted as “1” if it produced at least one similarity hit to any active reference molecule, and as “0” otherwise. The DCS therefore represents the sum of these binary indicators, corresponding to the number of descriptor families that identified at least one similarity match, independent of how many individual reference molecules triggered the hit within a descriptor. A DCS value of 7 indicates that all descriptor types identified at least one similar active molecule, whereas a value of 0 indicates that none of the descriptors produced any similarity matches.

A comparable analysis was performed to assess the similarity of set E against the 35 molecules comprising the inactive reference set (set I). Using the same similarity threshold (0.85) and the identical panel of descriptor types, an analogous metric, referred to as the global inactivity score (GI), was computed for each molecule in set E.

To integrate information from both similarity analyses, we introduced a heuristic composite ranking metric referred to as the S score. This metric combines the GA value, which reflects similarity to the active reference molecules, with the GI score, which captures similarity to the inactive set. The S score is defined as:

S = GA/(1 + GI)(1)

This formulation increases the weight of similarity to active compounds while proportionally reducing the contribution of molecules that also resemble inactive references. The constant term in the denominator prevents division by zero and provides a gradual penalty as GI increases. As a result, compounds with high GA and low GI values receive the highest S scores, offering a straightforward and interpretable way to prioritize molecules based on their position within both active and inactive chemical space. The S score was therefore used as a preselection criterion to define a subset of compounds for further evaluation, referred to as the Prioritized Similarity Set (PS set). Because the active and inactive reference sets contained different numbers of compounds (16 and 35, respectively), the absolute GA and GI values may be influenced by reference-set size; therefore, the S score was used only as a heuristic preselection criterion.

### 2.3. ADMET Prediction

ADMET profiling of the 140 structures in the PS set was carried out using ADMETlab 3.0, an in silico platform that provides binary or continuous probability predictions for absorption, distribution, metabolism, excretion, and toxicity (ADMET)-related properties [25]. The predicted parameters included physicochemical descriptors, absorption-related features, distribution metrics, metabolism-associated liabilities, and multiple toxicity endpoints.

Blood–brain barrier (BBB) permeability predictions were applied as an initial filtering criterion. Compounds with predicted BBB values below 0.5 were excluded, as they were considered unlikely to reach sufficient concentrations in the central nervous system (CNS) [26].

For compound prioritization, a heuristic composite drug-likeness score (DLK) was constructed. The DLK score combined four rule-based filters: Lipinski, Pfizer, GSK, and the Golden Triangle, each capturing complementary physicochemical and developability constraints relevant to orally bioavailable small-molecule drugs [27]. Compliance with each rule was assessed independently and encoded as a binary variable (1 = compliant, 0 = non-compliant). The DLK score was calculated as the unweighted sum of the four criteria, yielding values ranging from 0 to 4.

In parallel, a heuristic composite toxicity score (TOX) was generated to integrate multiple predicted toxicity liabilities into a single metric. The TOX score was based on four in silico toxicity endpoints: drug-induced liver injury (DILI), AMES mutagenicity, carcinogenicity, and genotoxicity, as implemented in ADMETlab 3.0. For each endpoint, predicted probabilities were evaluated using a threshold of 0.5. Probabilities equal to or above this threshold were classified as positive (toxic) and encoded as 1, whereas probabilities below 0.5 were classified as negative (non-toxic) and encoded as 0. The TOX score was calculated as the sum of the four endpoint values, resulting in scores ranging from 0 to 4, with higher scores indicating increased predicted toxicological burden.

### 2.4. Molecular Docking Study

The cryo-EM structure of the human β3 homopentameric GABA-A receptor in complex with histamine (PDB ID: 9FEU [28]) and human extrasynaptic α4/β3/δ GABA-A receptor in complex with GABA and histamine (PDB ID: 7QN7 [29]) were downloaded from the RCSB Protein Data Bank. The β3 homopentameric structure was also considered since the known active and inactive molecules were experimentally tested on this specific configuration [23]. Protein preparation consisted of removal of non-essential crystallographic waters and solvent molecules, as well as addition of hydrogen atoms. Protonation states were assigned for physiological pH of 7.4, followed by energy minimization with YASARA2 forcefield within YASARA Structure software (version 25.1.13) [30].

3D conformers for the virtual E library and for the 51 known compounds (active and inactive) were generated in DataWarrior. Ligands were protonated at physiological pH and underwent energy-minimization using the MMFF94s+ force field in order to obtain a single low-energy conformation per compound. The same protocol was also applied for the known active and inactive molecules.

Docking simulations were performed with AutoDock Vina 1.1.2 [31] as implemented in YASARA software. The search space was defined as a 25 × 25 × 25 Å gridbox, centered on the co-crystallized histamine, extended to fully encompass the allosteric binding pocket and its immediate surrounding residues. For each ligand, docking was performed to generate up to 12 poses, which were subsequently ranked according to their predicted binding energies. Binding poses and key receptor–ligand contacts were inspected and illustrated using BIOVIA Discovery Studio Visualizer (BIOVIA, Discovery Studio Visualizer, Version 17.2.0, Dassault Systèmes, 2016, San Diego, CA, USA).

For docking method validation, the crystallographic histamine was re-docked using the same protocol, and the RMSD (root mean square deviation) between docked and experimental poses was calculated to check that the docking setup could reproduce the reference binding mode. Moreover, the known active and inactive compounds were also docked into the histamine binding site for further validation. Receiver operating characteristic (ROC) curves and area under the curve (AUC) values were derived from the predicted binding energies (ΔG, kcal/mol) and ligand efficiencies (LE = −ΔG/Nheavy, where Nheavy represents the number of heavy atoms), and Mann–Whitney U tests were applied to assess whether score distributions differed significantly between active and inactive molecules. The binding efficiency index (BEI) was calculated as BEI = pKd/(MW/1000), where MW is the molecular weight in Da, whereas the surface efficiency index (SEI) was calculated as SEI = pKd/(PSA/100), where PSA is the polar surface area in Å^2^. Lipophilic ligand efficiency was calculated as LLE = pKd − logP, and ligand efficiency-dependent lipophilicity as LELP = logP/LE [32].

Only 10 out of the 16 active compounds were kept for statistical analysis since the docking algorithm could not predict plausible binding poses or significant interactions with key residues involved in histamine binding (e.g., Asp43, Glu155, or Gln64) for 6 ligands.

The virtual screening was performed using the GABA-A receptor structure that yielded the most plausible predictions of binding poses of known active molecules.

### 2.5. Molecular Dynamics Simulations and Free Binding Energy Calculation

Molecular dynamics (MD) simulations were performed to further evaluate the stability of selected protein–ligand complexes. Three compounds, CHEMBL1202320, CHEMBL521996, and CHEMBL3236582, were selected from the 13 prioritized hits based on their complementary similarity, ADMET, docking, and ligand-efficiency profiles. CHEMBL1202320 showed the highest GA value (13) and a high DCS (6), CHEMBL521996 combined high similarity scores with favorable predicted BBB permeability, while CHEMBL3236582 showed GI = 0, a high S score (8), favorable BBB permeability, and a binding mode reproducing key histamine interactions. Considering the computational cost of MD simulations, these three compounds were selected for further evaluation. In addition to the ligand-bound systems, two reference systems were included: the ligand-free GABA-A receptor (apo form) as a negative control and the experimentally determined GABA-A/histamine complex as a positive control. All simulations were carried out using YASARA Structure. The protein–ligand complexes were solvated in an explicit water box and simulated at physiological pH (7.4) under isothermal–isobaric (NPT) conditions. System neutralization was achieved by adding Na^+^/Cl^−^ ions to approximate physiological ionic strength [33]. Prior to production runs, each system underwent energy minimization using a combination of steepest descent and simulated annealing to eliminate unfavorable steric contacts. Phosphatidylethanolamine lipid bilayers were generated automatically to embed the receptor systems. During system setup, the protein–ligand complexes were initially scaled to 0.9 in order to facilitate membrane insertion and overlapping lipid molecules were removed. The scaling factor was then gradually restored during a short equilibration run performed in vacuo at 298 K. Protein coordinates were incrementally rescaled by a factor of 1.02 at 200 fs intervals, while membrane atoms were allowed to relax and adapt to the presence of the protein.

The AMBER14 force field was applied to the protein, Lipid17 for the lipid bilayer, while ligand parameters were assigned using GAFF2 with AM1-BCC partial charges. Water molecules were modeled using the TIP3P model. Van der Waals interactions were computed using a cutoff distance of 8 Å, and long-range electrostatic interactions were treated using the particle mesh Ewald (PME) method. Temperature and pressure were maintained at 298 K and 1 atm, respectively. The equations of motion were integrated using a multiple time-step algorithm, with a time step of 2.5 fs for bonded interactions and 5 fs for non-bonded interactions. Each system was simulated for a total duration of 100 ns. Binding free energies (kcal/mol) were estimated using the Molecular Mechanics/Poisson–Boltzmann Surface Area (MM/PBSA) approach, excluding the entropic contribution, to enable a comparative assessment of ligand binding stability throughout the simulation trajectory [34,35].

## 3. Results

### 3.1. Ligand Datasets

The final ligand library comprised an evaluation set (set E) consisting of 5641 unique molecules. For comparative molecular similarity analysis, two reference sets were defined: an active set (set A), composed of 16 compounds with known negative allosteric modulator (NAM) activity on GABA-A receptors, and an inactive set (set I), consisting of 35 compounds lacking this activity. These reference sets were used exclusively as input data for structure–activity relationship (SAR) analysis and for validation of the molecular docking protocol.

### 3.2. Structure Analysis

A total of 791 molecules received a GA greater than zero, indicating similarity to at least one active reference molecule for at least one descriptor type. This group was designated the active-hit set (AH). The maximum GA observed was 13, and nearly half of the AH molecules (50.8%) had a score of 1, reflecting only weak similarity to the compounds in set A.

Only two compounds reached the maximum DCS value of 7, and among the 791 molecules with at least one similarity hit, 275 had a DCS of 2 or higher, reflecting agreement across multiple descriptor families.

In the E set, a total of 2134 molecules received a GI greater than zero, indicating similarity to at least one inactive reference molecule according to at least one descriptor. The maximum GI observed was 19. The GI values showed a broad, right-skewed distribution (mean 1.45, standard deviation 2.81), indicating that while many molecules displayed minimal similarity to the inactive reference set, a substantial number formed a long tail of higher GI values.

The distribution of S values across the E set was right-skewed (mean 1.01, median 0.64, standard deviation 1.16), indicating that the majority of molecules have relatively low S scores and only a minority showed high similarity to the active reference set combined with low similarity to the inactive set. For scaffold-oriented prioritization, an operational cutoff of S ≥ 2 was selected. This value lies approximately 0.85 standard deviations above the mean and retained 140 compounds, corresponding to approximately 18% of the 791 molecules with GA > 0. This subset was designated the PS set.

Visual inspection of the PS structures also revealed several recurring molecular features, including at least one aromatic ring, at least three electronegative atoms (usually including two nitrogen atoms), and a minimum of three hydrogen-bond acceptor sites. The compounds can be classified into four major categories: imidazole derivatives, 2,4-diaminopyrimidine derivatives, indole-2-carboxamide derivatives, and a final group comprising structurally diverse molecules. The imidazole derivative group contains 57 molecules, the majority of which (35 compounds) incorporate an isothiourea scaffold. Within this subgroup, molecules in which the sulfur atom is linked to the imidazole ring through an alkyl chain tend to exhibit higher GA scores than analogues with an unsubstituted sulfur atom. The 2,4-diaminopyrimidine category includes 27 compounds, 25 of which carry an ethylenediamine substituent at the 4-position, either in an acyclic form or incorporated into a cyclic structure. The third category consists of 39 indole-2-carboxamide derivatives. The remaining category contains 17 molecules, of which 11 share a common piperazine scaffold.

The PS set included two approved drugs, sertindole and tolvaptan, both with modest similarity values (GA = 2; S = 2). Sertindole (ATC code N05AE04) is an atypical antipsychotic acting primarily on dopaminergic and serotonergic receptors; and tolvaptan (ATC code C03XA01) is a V_2_-receptor antagonist used clinically as an aquaretic agent. ADMET analysis predicted a high probability of blood–brain barrier permeability for sertindole (BBB = 0.999), whereas tolvaptan showed a very low predicted BBB permeability (BBB = 0.008).

The highest-ranking molecules from the PS set, based on their GA scores, are presented in Table 2. For each compound, the ChEMBL identifier, chemical structure, GA, GI, S, and DCS are reported.

### 3.3. Predicted ADMET Profiles

BBB permeability predictions indicated that 104 compounds exceeded the 0.5 threshold, suggesting a substantial proportion of the dataset possesses the physicochemical characteristics required for adequate CNS exposure.

Analysis of the DLK score distribution showed that 82 compounds achieved a perfect score of 4, while 36 compounds exhibited a single deviation from the preferred criteria (score = 3). Twenty compounds had a score of 2, and two compounds had a score of 1. No compounds received a score of 0. For subsequent analyses, only compounds with DLK scores equal to or greater than 3 were retained, as this threshold reflects acceptable compliance with established oral drug-likeness criteria.

Evaluation of individual DLK components revealed full compliance with the Lipinski rule across the dataset, indicating favorable oral drug-likeness in terms of molecular weight, lipophilicity, and hydrogen bonding capacity. The highest number of deviations was observed for the Pfizer criterion, with 44 compounds failing to meet this rule, which emphasizes an optimal balance between lipophilicity and polar surface area to reduce attrition risks associated with toxicity and off-target promiscuity.

The distribution of TOX scores demonstrated marked variability in predicted toxicity liabilities. Five compounds exhibited a TOX score of 0, indicating no predicted toxicological concerns across the evaluated endpoints, while 17 compounds had a score of 1. Scores of 2 and 3 were observed for 38 and 34 compounds, respectively, and 46 compounds reached the maximum TOX score of 4.

Following sequential filtering based on BBB permeability and DLK score, the PS set was reduced to 88 compounds. Final prioritization was achieved by selecting compounds with TOX scores of 2 or lower, resulting in 44 candidates with predicted favorable pharmacokinetic and safety profiles.

### 3.4. Molecular Docking

#### 3.4.1. Docking Validation

**Figure 1:**
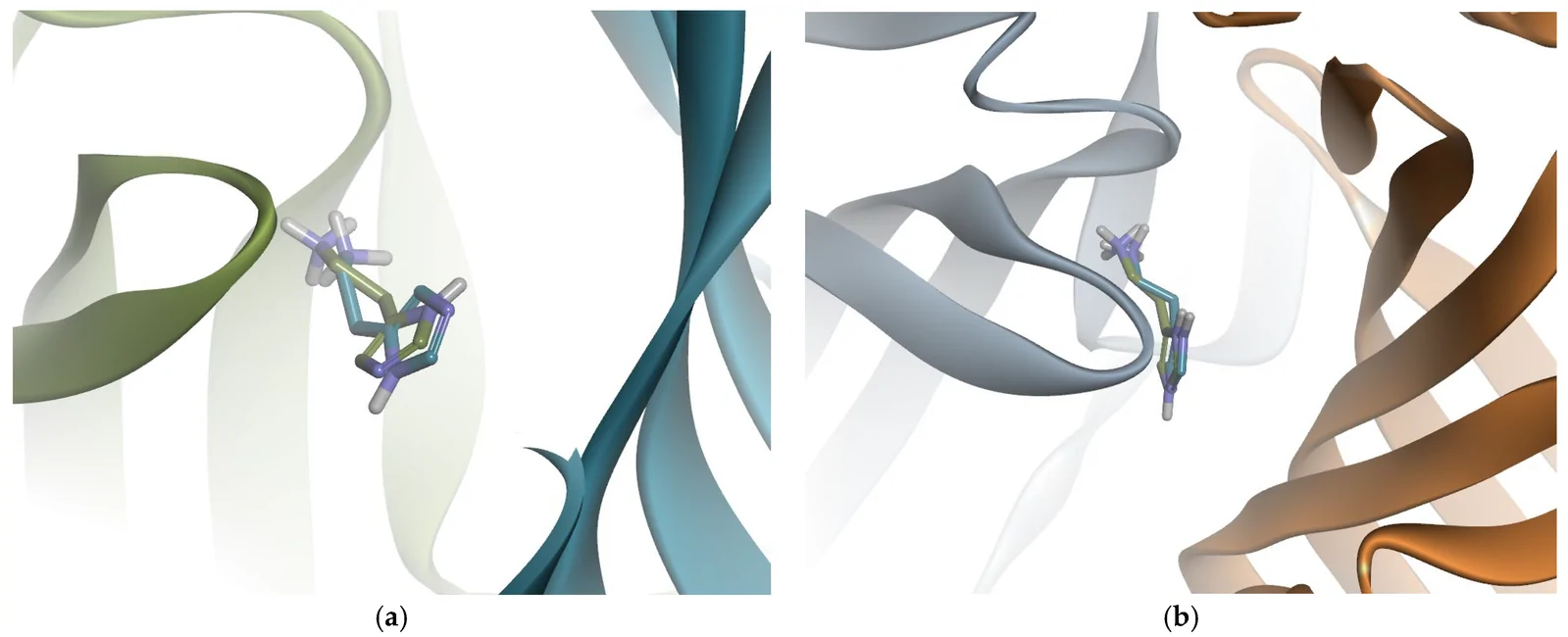
Superposition of redocked histamine conformations (blue) over experimentally determined histamine conformation (green). (**a**) β3 homopentameric GABA-A receptor (PDB ID: 9FEU); (**b**) extrasynaptic heteropentameric α4/β3/δ configuration (PDB ID: 7QN7).

For the β3 homopentameric GABA-A receptor (PDB ID: 9FEU), superposition of the docked histamine conformation onto the experimental structure resulted in an RMSD value of 1.96 Å (Figure 1a), indicating satisfactory reproduction of the binding pose within the active site. In the case of the extrasynaptic heteropentameric α4/β3/δ configuration (PDB ID: 7QN7), a RMSD of 0.78 Å was obtained (Figure 1b). The predicted binding energies for histamine were −5.11 kcal/mol for the homopentameric configuration (LE = 0.639) and −4.49 kcal/mol for the extrasynaptic configuration (LE = 0.561).

Binding modes of compounds with known activity (active set A) were further evaluated by visual inspection, considering pharmacophore orientation and predicted receptor–ligand interactions. The β3 homopentameric receptor generated plausible binding poses for 10 out of 16 active compounds, whereas the extrasynaptic configuration yielded only five poses deemed reasonable. Based on these results, subsequent analyses and virtual screening were carried out exclusively using the β3 homopentameric GABA-A receptor. The predicted binding energies and ligand efficiency values for the 10 active reference compounds are shown in Table 3.

**Figure 2:**
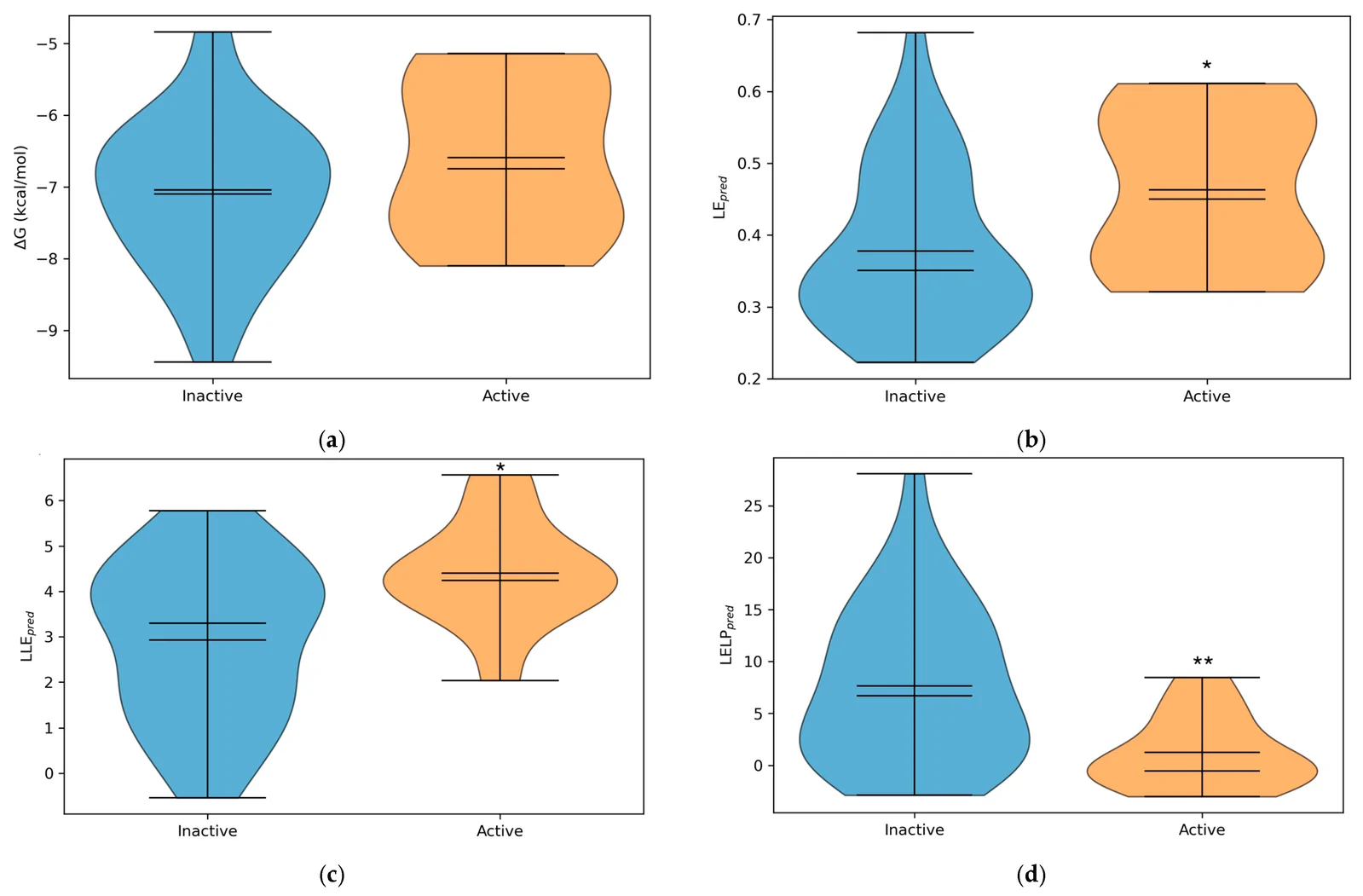

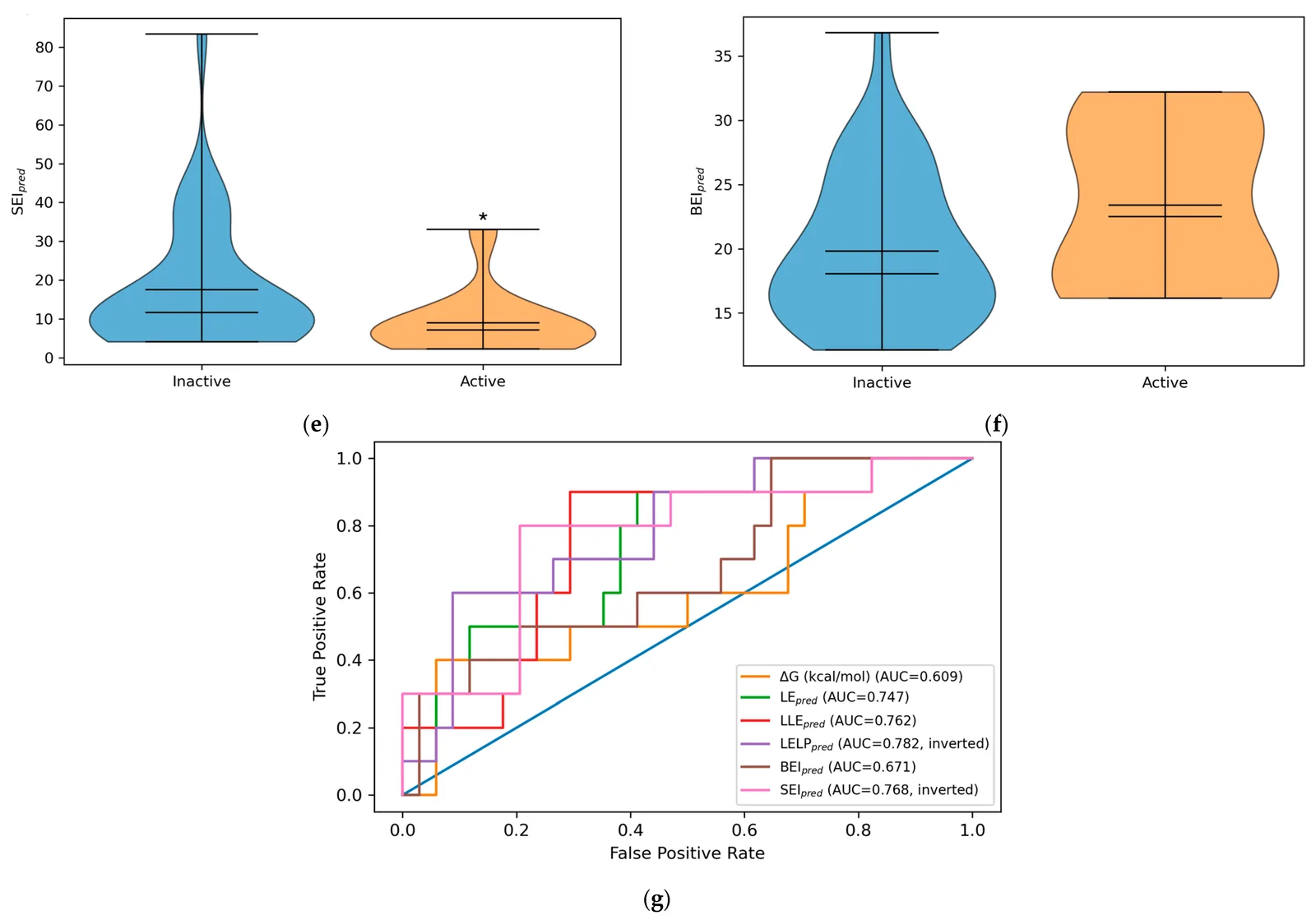
Mean ± SD values for active and inactive ligands showing predicted: (**a**) ΔG; (**b**) LE; (**c**) LLE; (**d**) LELP; (**e**) SEI; (**f**) BEI; (**g**) ROC curves based on all predicted parameters. * *p* < 0.05, ** *p* < 0.01. ΔG—binding energy; LE—ligand efficiency; BEI—binding efficiency index; surface efficiency index; LLE—lipophilic ligand efficiency; LELP—ligand efficiency-dependent lipophilicity.

To assess the ability of docking-derived descriptors to discriminate between active (*n* = 10) and inactive (*n* = 34) histaminergic ligands, predicted binding energy and several ligand efficiency metrics were analyzed. The mean predicted binding energy was comparable between the two classes (−6.59 ± 1.11 kcal/mol for active ligands vs. −7.10 ± 1.07 kcal/mol for inactive compounds), with no statistically significant difference observed (Mann–Whitney U test, *p* = 0.307, Figure 2a). In contrast, active ligands exhibited significantly higher predicted ligand efficiency (LE; 0.463 ± 0.111 vs. 0.378 ± 0.114, *p* = 0.019, Figure 2b) and lipophilic ligand efficiency (LLE; 4.40 ± 1.21 vs. 2.92 ± 1.73, *p* = 0.013, Figure 2c). Active compounds also showed significantly lower ligand efficiency-dependent lipophilicity (LELP; 1.28 ± 3.55 vs. 7.64 ± 7.62, *p* = 0.007, Figure 2d) and surface efficiency index (SEI; 8.99 ± 8.86 vs. 17.50 ± 16.78, *p* = 0.011, Figure 2e), whereas the binding efficiency index (BEI) did not differ significantly between the two groups (23.41 ± 6.53 vs. 19.81 ± 5.96, *p* = 0.107, Figure 2f). ROC analysis corroborated these findings. Predicted binding energy displayed limited discriminative ability (ROC AUC = 0.61, Figure 2g), whereas LE (ROC AUC = 0.75), LLE (ROC AUC = 0.76), LELP (ROC AUC = 0.78), and SEI_pred_ (ROC AUC = 0.77) exhibited good discrimination between active and inactive ligands. BEI showed only moderate predictive performance (ROC AUC = 0.67). Among the evaluated descriptors, LELP demonstrated the highest discriminative power, closely followed by SEI and LLE, suggesting that lipophilicity- and surface-normalized efficiency metrics provide greater value than docking score alone for prioritizing histaminergic ligands during virtual screening.

However, LE and LLE combined favorable discriminative performance with an intuitive medicinal chemistry interpretation, making them the most suitable metrics for prioritizing candidate histaminergic ligands during virtual screening. Although LELP achieved the highest ROC AUC, its inverse relationship with activity reflects that lower values denote greater lipophilic efficiency. Moreover, SEI also showed good discrimination only after inversion, likely reflecting the relatively high polar surface area required for active histaminergic ligands, limiting its general applicability as a prospective screening metric. Therefore, LE and LLE were further used to prioritize compounds undergoing virtual screening.

#### 3.4.2. Virtual Screening

For the E set subjected to virtual screening (*n* = 5641), the predicted binding energy showed an average of −8.13 ± 0.88 kcal/mol, with values ranging from −11.86 to −4.89 kcal/mol. The ligand efficiency had a mean value of 0.328 ± 0.064, the observed range being 0.145–0.688. Compared to histamine, only one compound exceeded the LE value, indicating that most new compounds have a lower binding efficiency than the endogenous ligand.

Next, the 44 compounds from the PS set selected based on the SAR analysis and the ADMET profile were further analyzed. The predicted binding energy values showed an average of −7.69 kcal/mol, with a standard deviation of 1.12, indicating a moderate variability of the parameter, and the range of variation is between −10.62 and −4.76. The LE parameter had a mean value of 0.373, with extreme values ranging from 0.213 to 0.627 and a standard deviation of 0.078.

Of the 44 compounds with a favorable ADMET profile, 13 structures were prioritized according to ligand efficiency values: (Table 4). Of these, 3 hit molecules were selected for the analysis of predicted molecular interactions and for the subsequent molecular dynamics study: CHEMBL1202320 (3-(1H-imidazol-5-yl)propyl N′-[2-(4-methylphenyl)ethyl]carbamimidothiate), CHEMBL521996 (6-[(3R)-3-(methylamino)pyrrolidin-1-yl]-3,5-diazatricyclo[9.4.0.0^2,7^]pentadeca-1(15),2(7),3,5,11,13-hexaene-4-amine), and CHEMBL3236582 (4-[(3R)-3-(methylamino)pyrrolidin-1-yl]-5,6,7,8-tetrahydroquinazolin-2-amine).

**Figure 3:**
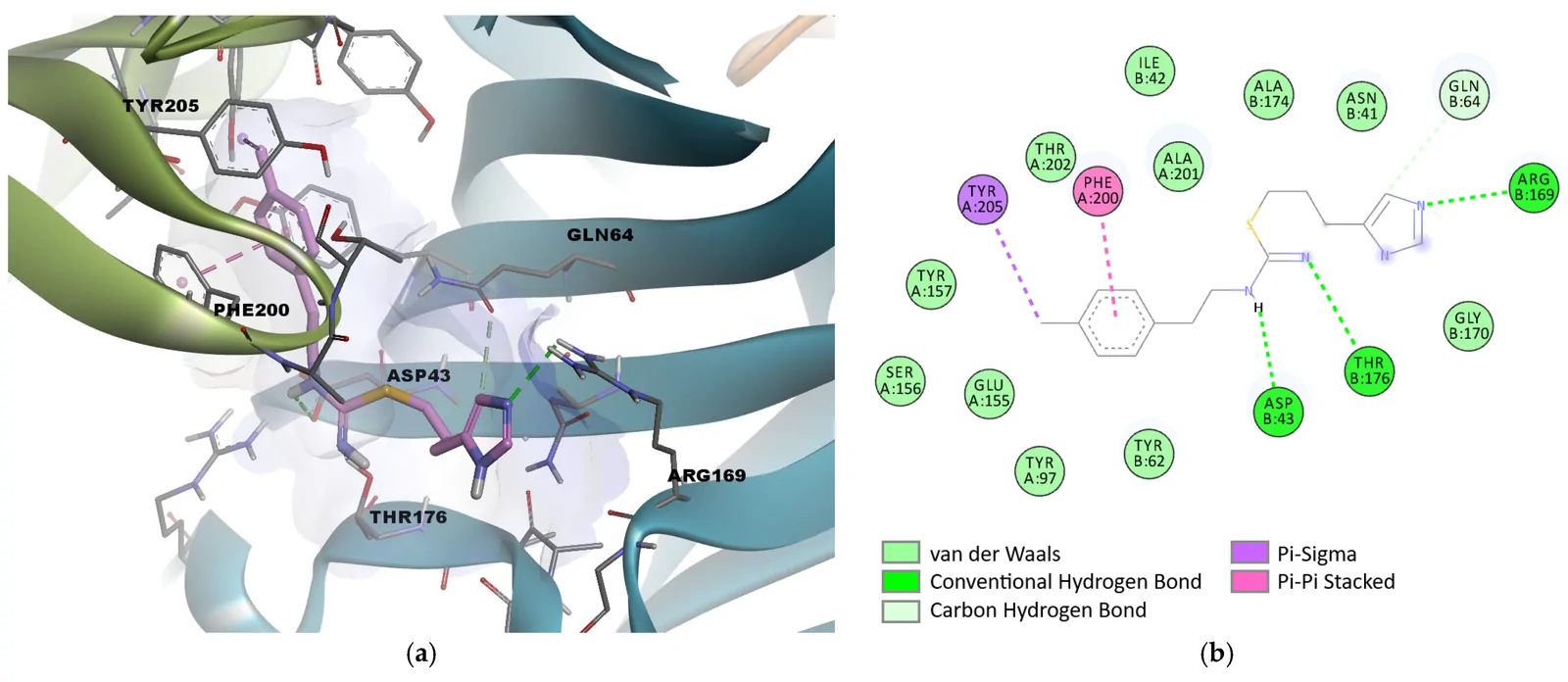
Predicted conformation of CHEMBL1202320 in the allosteric site of homopentameric GABA-A (**a**) and 2D diagram of predicted molecular interactions (**b**).

CHEMBL1202320 exhibited a binding mode compatible with the allosteric site of the GABA-A receptor, being stabilized by a hydrogen bond network with Arg169, Thr176 and Asp43. Although the direct electrostatic interaction with Glu155, characteristic of histamine, is not conserved, the compound partially reproduces the aromatic pharmacophore of the endogenous ligand through π–σ interactions with Tyr205 and Phe200, residues also involved in histamine stabilization. The preservation of these key contacts suggests a favorable orientation of the ligand at the binding site (Figure 3).

**Figure 4:**
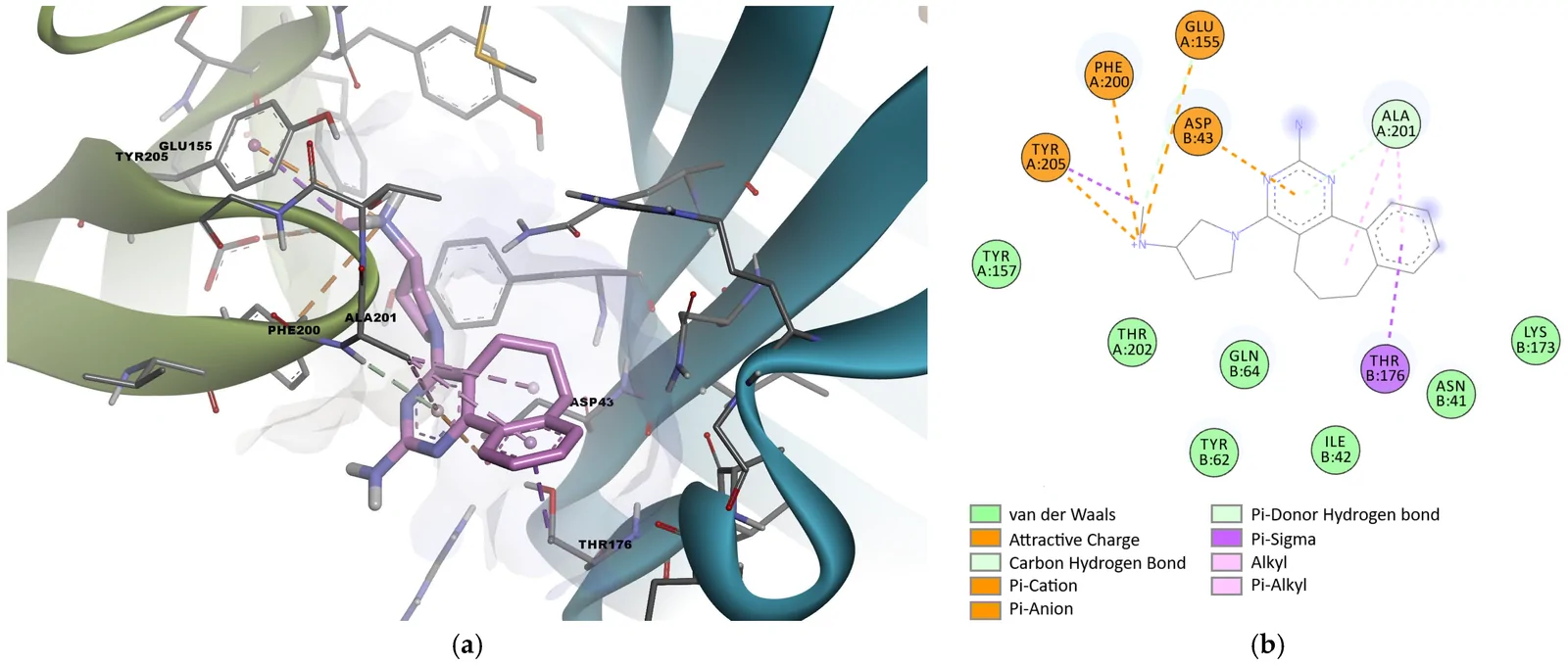
Predicted conformation of CHEMBL521996 in the allosteric site of homopentameric GABA-A (**a**) and 2D diagram of predicted molecular interactions (**b**).

CHEMBL521996 reproduced several key histamine interactions in the specific binding site, being electrostatically anchored through a salt bridge with Glu155, considered highly relevant for the recognition of the endogenous ligand. The compound also participated in the formation of a carbon-hydrogen bond and π–cation-type electrostatic interactions with Phe200 and Tyr205, as well as π–anion interactions with Asp43. In addition, hydrophobic and aromatic contributions involving Thr176, Tyr205, and Ala201 support favorable ligand positioning at the binding site (Figure 4). Overall, the preservation of the critical interaction with Glu155 and major aromatic contacts suggests that CHEMBL521996 closely mimics histamine, justifying its selection as a priority hit molecule for further studies.

**Figure 5:**
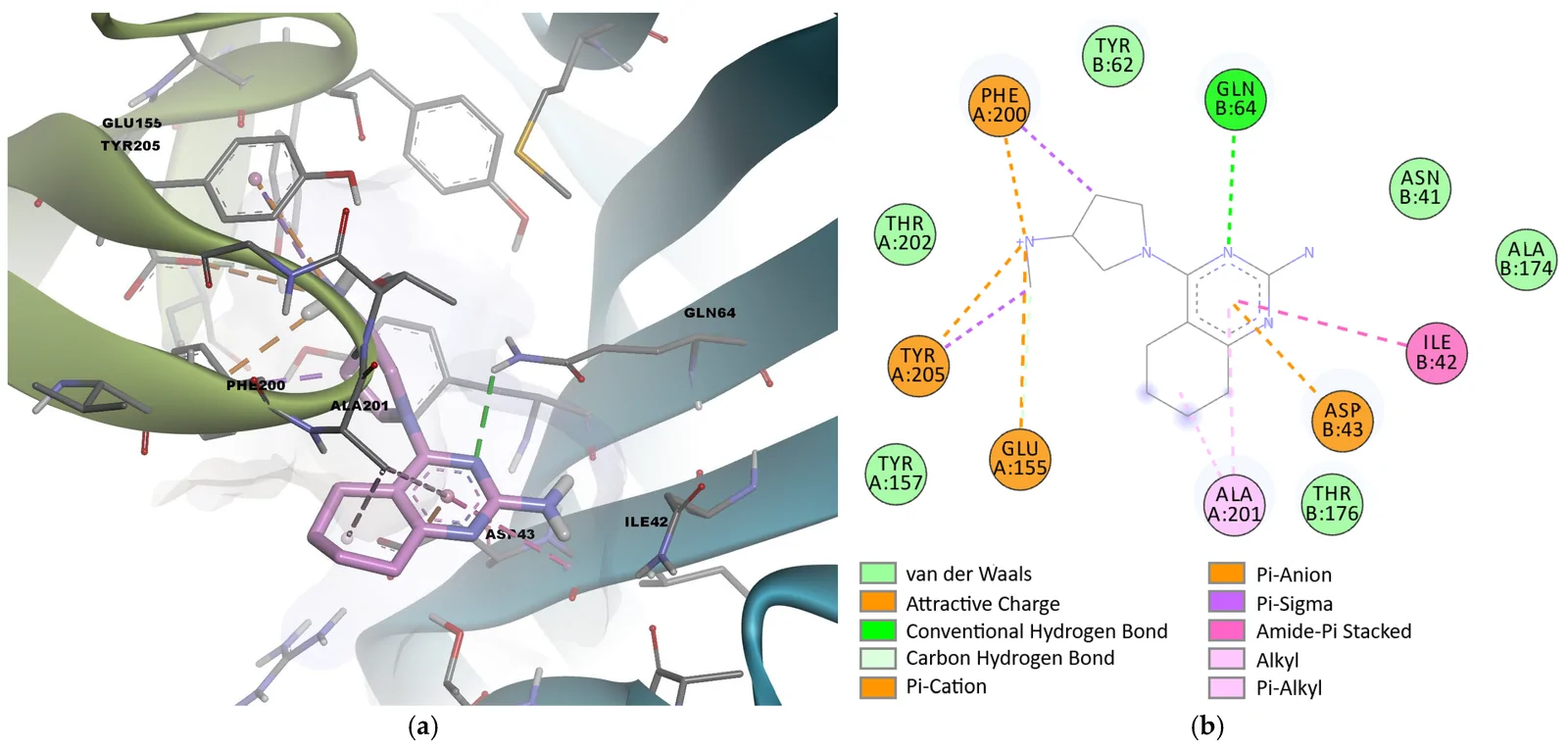
Predicted conformation of CHEMBL3236582 in the allosteric site of homopentameric GABA-A (**a**) and 2D diagram of predicted molecular interactions (**b**).

CHEMBL3236582 has a binding mode similar to that of histamine, being electrostatically anchored through a salt bridge with Glu155. Additional hydrogen bonds with Gln64 were observed, as well as π–cation electrostatic interactions with Phe200 and Tyr205 and π–anion with Asp43. Hydrophobic and aromatic interactions with Phe200, Tyr205 and Ala201, together with π-amide contacts with Ile42, contribute to a favorable orientation of the ligand in the histamine site (Figure 5). Overall, the conservation of key interactions with Glu155, Phe200 and Tyr205 indicates effective histamine mimicking and supports the selection of CHEMBL3236582 as a promising hit molecule for further steps.

### 3.5. Molecular Dynamics

**Figure 6:**
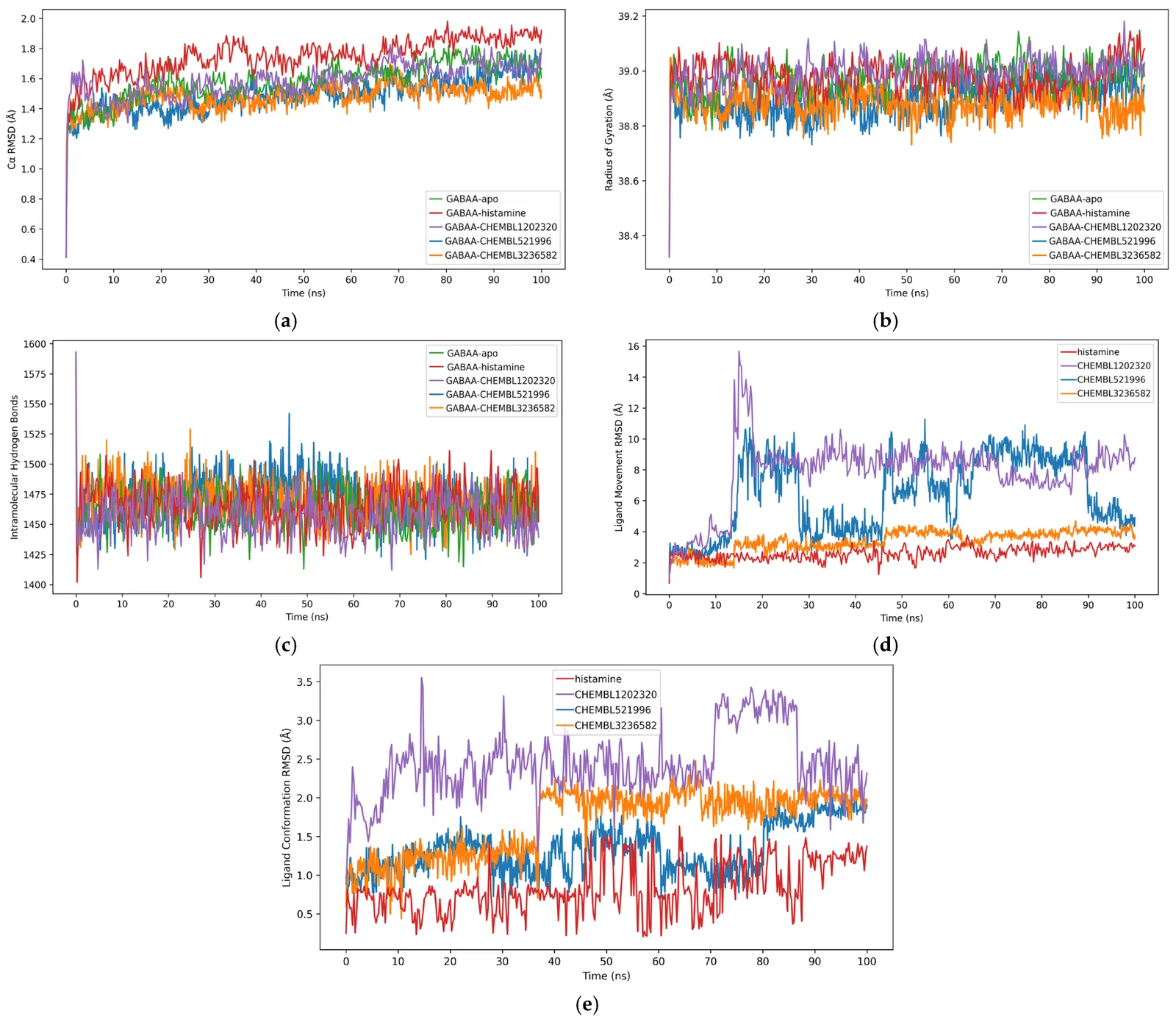
MD results after 100 ns simulation time. (**a**) RMSD values of protein Cα atoms; (**b**) radius of gyration (Rg) variation; (**c**) total number of intramolecular hydrogen bonds; (**d**) RMSD values for ligand movement within the binding site; (**e**) RMSD values for ligand conformations after superposition on initial snapshot.

MD simulations revealed that all systems reached structural equilibration within the first 1 ns, as indicated by stabilization of Cα RMSD values (Figure 6a). The apo GABA-A receptor displayed a stable backbone conformation with a mean RMSD of 1.59 ± 0.12 Å after equilibration. Binding of histamine induced a modest increase in backbone deviations (1.75 ± 0.11 Å), consistent with minor ligand-induced conformational adjustments while preserving the overall structural integrity of the receptor. Among the investigated hit compounds, CHEMBL1202320 produced an RMSD profile nearly identical to that of the apo receptor (1.59 ± 0.10 Å), indicating excellent structural stability throughout the simulation. CHEMBL521996 and CHEMBL3236582 exhibited even lower average RMSD values (1.50 ± 0.11 Å and 1.48 ± 0.07 Å, respectively), with CHEMBL3236582 showing the smallest fluctuations among all ligand-bound systems. No progressive increase in RMSD or abrupt structural transitions were observed for any complex over the 100 ns production phase, indicating that all three selected hit compounds maintain stable interactions with the GABA-A receptor. These results demonstrate that the docking-derived binding poses remain structurally stable during extended molecular dynamics simulations and that ligand binding does not induce significant backbone destabilization of the receptor.

Radius of gyration (Rg) analysis demonstrated that the global compactness of the GABA-A receptor was well preserved throughout the 100 ns simulations (Figure 6b). Following a short equilibration period during approximately the first 1 ns, all systems reached stable Rg plateaus with only minor fluctuations, indicating maintenance of the overall protein fold during the production phase. The apo receptor and the histamine-bound complex exhibited nearly identical average Rg values (38.98 ± 0.06 Å and 38.98 ± 0.06 Å, respectively), confirming that histamine binding does not alter the global structural compactness of the receptor. Similarly, the complex containing CHEMBL1202320 remained highly stable, displaying a comparable mean Rg of 39.00 ± 0.05 Å. In contrast, complexes formed with CHEMBL521996 and CHEMBL3236582 exhibited slightly lower average Rg values (38.90 ± 0.06 Å and 38.89 ± 0.05 Å, respectively), suggesting a marginally more compact receptor conformation while maintaining equally low fluctuations throughout the simulation. Importantly, no progressive increase in Rg or signs of structural expansion were observed for any ligand-bound complex over the 100 ns trajectory. Therefore, binding of the selected hit compounds preserves the overall structural integrity and compactness of the GABA-A receptor, further supporting the stability of the docking-derived binding modes.

Analysis of intramolecular hydrogen bonds revealed that all simulated systems underwent rapid equilibration during approximately the first 1 ns, after which a stable hydrogen-bonding network was maintained throughout the remainder of the 100 ns trajectories (Figure 6c). The apo GABA-A receptor exhibited an average of 1462 ± 16 intramolecular hydrogen bonds following equilibration, reflecting the intrinsic stability of the receptor structure. The histamine-bound complex showed a nearly identical hydrogen-bond count (1465 ± 17), indicating that binding of the endogenous ligand does not perturb the internal hydrogen-bonding network. Similarly, complexes formed with CHEMBL1202320, CHEMBL521996, and CHEMBL3236582 maintained stable hydrogen-bond populations of 1457 ± 16, 1470 ± 18, and 1468 ± 16, respectively. Although CHEMBL521996 and CHEMBL3236582 exhibited slightly higher average hydrogen-bond counts than the apo and histamine-bound systems, these differences were small and remained within the range of normal thermal fluctuations observed during the simulations. Importantly, none of the ligand-bound complexes displayed a progressive loss of intramolecular hydrogen bonds or evidence of hydrogen-bond network disruption over the 100 ns production phase. These findings showed that binding of the selected hit compounds preserved the internal hydrogen-bond architecture of the GABA-A receptor and further supported the structural stability of the docking-derived complexes during extended molecular dynamics simulations.

Ligand movement RMSD analysis showed that all ligands underwent an initial positional adjustment within approximately the first 1 ns, followed by stabilization inside the binding pocket for the remainder of the 100 ns simulations (Figure 6d). Histamine displayed the lowest positional fluctuations throughout the trajectory, maintaining a mean ligand RMSD of 2.63 ± 0.39 Å, consistent with a stable reference binding mode. Among the screened compounds, CHEMBL3236582 exhibited the most stable binding behavior, with a mean RMSD of 3.41 ± 0.67 Å, remaining close to its initial docking pose during the entire simulation. In contrast, CHEMBL521996 adopted a different but stable binding orientation after the initial equilibration, resulting in a higher average ligand RMSD (6.44 ± 2.39 Å) without evidence of progressive drift or ligand dissociation. CHEMBL1202320 showed the greatest positional mobility, reaching a mean RMSD of 7.92 ± 2.08 Å and exhibiting larger fluctuations throughout the simulation, indicative of continuous repositioning within the binding cavity. Nevertheless, no abrupt increases in RMSD or complete unbinding events were observed for any ligand over the 100 ns trajectory, suggesting that all compounds remained associated with the receptor. Overall, ligand movement analysis identified CHEMBL3236582 as the most positionally stable hit, whereas CHEMBL1202320 and, to a lesser extent, CHEMBL521996 displayed increased mobility within the binding pocket while maintaining persistent receptor interactions.

Ligand conformational RMSD analysis showed that all ligands reached conformational equilibrium within approximately the first 1 ns and remained structurally stable throughout the 100 ns simulations (Figure 6e). Histamine exhibited the lowest conformational deviations, maintaining a mean RMSD of 0.83 ± 0.33 Å, consistent with its compact structure and limited internal flexibility. Among the investigated hit compounds, CHEMBL521996 displayed the greatest conformational stability, with a mean RMSD of 1.32 ± 0.31 Å, indicating only minor internal rearrangements while bound to the receptor. CHEMBL3236582 showed slightly greater conformational flexibility, reaching an average RMSD of 1.68 ± 0.41 Å, although its trajectory remained stable throughout the simulation without evidence of progressive conformational drift. In contrast, CHEMBL1202320 exhibited the highest conformational RMSD (2.45 ± 0.43 Å), reflecting increased internal flexibility relative to the other ligands while still maintaining a well-defined conformational ensemble after equilibration. Importantly, none of the ligands displayed abrupt increases in conformational RMSD or signs of unfolding during the production phase, indicating that their internal geometries remained stable despite differences in conformational plasticity. Overall, these results suggested that all three hit compounds preserved stable bound conformations over the 100 ns simulations, with CHEMBL521996 exhibiting the highest conformational rigidity, CHEMBL3236582 displaying intermediate flexibility, and CHEMBL1202320 remaining the most conformationally dynamic ligand.

Binding free energies were estimated using the MM/PBSA approach based on the production phase of the 100 ns molecular dynamics simulations, excluding the initial 1 ns equilibration period. Among the investigated hit compounds, CHEMBL3236582 exhibited the most favorable binding free energy (−280.8 kcal/mol), slightly exceeding both the reference ligand and the other screened compounds, indicating the strongest energetic stabilization of the receptor–ligand complex. CHEMBL521996 displayed a similarly favorable minimum binding free energy (−276.9 kcal/mol), also surpassing that of histamine, whereas CHEMBL1202320 reached a less favorable minimum value (−247.9 kcal/mol). These results are in good agreement with the molecular dynamics trajectory analyses, in which CHEMBL3236582 consistently exhibited the greatest positional stability and limited conformational flexibility, while CHEMBL521996 maintained stable binding despite undergoing a larger rigid-body reorientation within the binding pocket. Conversely, the comparatively weaker MM/PBSA profile of CHEMBL1202320 is consistent with its higher ligand movement and conformational RMSD values, suggesting a more dynamic binding mode. The 100 ns simulations identified CHEMBL3236582 as the most favorable ligand among the evaluated compounds, with CHEMBL521996 representing a second promising hit whose favorable binding free energy compensated for its increased positional mobility.

## 4. Discussion

In the present study, a comprehensive computational workflow integrating ligand-based similarity analysis, ADMET profiling, molecular docking, ligand efficiency assessment, and molecular dynamics simulations was applied to identify novel candidate ligands targeting the GABA-A receptor histamine-associated allosteric at the β3–β3 subunit interface. This multistep strategy progressively reduced a library of more than 5600 histaminergic ligands to a small number of structurally diverse candidates exhibiting favorable predicted ADMET properties, docking behavior, and dynamic stability. The use of histaminergic ligands as the source of the screening library represents an intentional chemical-space restriction. This strategy was selected because the target pocket recognizes histamine and because the experimentally characterized reference ligands available for this site are histaminergic compounds.

Previous experimental studies demonstrated that histamine and several histaminergic ligands can directly interact with GABA-A receptors, including β3-containing receptor configurations [15,17,23]. Moreover, combined mutagenesis, electrophysiological, and docking studies identified the β3–β3 interface as a histamine-binding region and highlighted residues such as Asp43 and Gln64 as relevant for ligand recognition [36]. The predicted interactions observed in the present study are therefore consistent with previous experimental and computational findings, although the pharmacological activity of the identified compounds remains to be experimentally confirmed.

The ligand-based prioritization strategy proved particularly effective because it considered both similarity to known active compounds and dissimilarity from inactive ligands. Rather than relying on a single molecular fingerprint, the descriptor consensus approach integrated complementary 2D and 3D representations of molecular structure, enabling identification of compounds sharing relevant pharmacophoric features despite substantial scaffold diversity. The resulting S score efficiently focused the screening campaign on molecules occupying chemical space associated with previously reported GABA-A receptor binders while reducing enrichment of chemotypes associated with the nonbinding reference set.

Inactive compounds showed slightly more favorable predicted binding energies than active ligands, although the difference was not statistically significant. This finding highlights the limited ability of docking scores to reproduce biological activity, as these values are influenced by ligand size, lipophilicity, and the number of receptor–ligand contacts. The low discriminative performance of predicted binding energy (ROC AUC = 0.61) further supports the use of ligand efficiency-related parameters as complementary criteria for compound prioritization. Although predicted binding energy showed only modest discriminative ability, LE, LLE, LELP, and SEI achieved ROC AUC values between approximately 0.75 and 0.78. Differences between predicted binding energy and LE rankings are expected, since LE normalizes binding energy to the number of heavy atoms. Therefore, larger ligands may show favorable docking scores but only moderate LE values. These parameters provide complementary information and do not necessarily rank compounds similarly.

Importantly, LELP and SEI required inversion because lower values were associated with biological activity, reflecting favorable lipophilic efficiency and reduced polar surface-normalized binding efficiency, respectively. From a practical perspective, however, LE and LLE represent the most useful descriptors for prospective virtual screening because they combine good predictive performance with straightforward medicinal chemistry interpretation. These observations further emphasize that docking scores alone frequently fail to rank ligands according to biological activity, whereas efficiency metrics normalize affinity by physicochemical properties and therefore may provide a more meaningful basis for hit prioritization.

Molecular dynamics simulations provided additional support for the structural stability of the selected receptor–ligand complexes. All systems rapidly reached equilibrium and maintained stable backbone RMSD, radius of gyration, and intramolecular hydrogen-bond networks throughout the production phase, indicating preservation of receptor structural integrity regardless of ligand binding. These findings indicate that occupation of the histamine-associated allosteric pocket does not induce large-scale conformational perturbations of the GABA-A receptor.

Analysis of ligand dynamics provided additional insight into the binding behavior of the prioritized compounds. CHEMBL3236582 consistently displayed the lowest ligand movement RMSD together with moderate conformational flexibility, indicating persistent occupation of the histamine binding pocket. In contrast, CHEMBL521996 underwent a substantially larger positional rearrangement while maintaining low conformational RMSD, suggesting that the ligand adopts an alternative but stable binding orientation through rigid-body reorganization rather than progressive destabilization. CHEMBL1202320 exhibited both the highest positional and conformational mobility, indicating a more dynamic interaction with the receptor despite remaining bound throughout the simulation. The favorable dynamic profile of CHEMBL3236582 may be related to its compact tetrahydroquinazoline scaffold and protonatable methylaminopyrrolidine substituent. This structure allows electrostatic anchoring to Glu155, hydrogen bonding with Gln64, and additional interactions with Asp43, Phe200, Tyr205, Ala201, and Ile42. The relatively constrained scaffold may also contribute to the lower ligand mobility observed during MD simulation compared with the more flexible CHEMBL1202320. Overall, these structural features may favor a stable orientation of CHEMBL3236582 within the histamine-associated pocket, although experimental confirmation is required.

MM/PBSA calculations were consistent with these dynamic observations. CHEMBL3236582 exhibited the most favorable binding free energy among all investigated ligands, followed closely by CHEMBL521996, whereas CHEMBL1202320 showed comparatively weaker energetic stabilization. The agreement between energetic analysis and molecular dynamics descriptors reinforces CHEMBL3236582 as the strongest computational candidate identified in the present study.

Several limitations should nevertheless be acknowledged. The validation dataset comprised only ten experimentally active ligands with reported affinities, limiting the statistical power of the analyses. Furthermore, MM/PBSA calculations were performed without explicit entropic contributions and therefore should primarily be interpreted comparatively rather than as estimates of absolute binding affinity. Finally, although the computational workflow successfully prioritized promising candidates, experimental electrophysiological characterization and binding studies remain essential to confirm their mechanism of action and pharmacological profile.

## 5. Conclusions

An integrated computational workflow combining ligand-based similarity analysis, ADMET prediction, molecular docking, and molecular dynamics simulations successfully identified promising candidate modulators targeting the histamine-associated allosteric pocket of the GABA-A receptor. The results demonstrate that ligand efficiency metrics, particularly LE and LLE, provide superior prioritization of active histaminergic ligands compared with docking score alone and therefore represent valuable complementary criteria for prospective virtual screening. Among the three prioritized compounds, CHEMBL3236582 consistently exhibited the most favorable overall profile, combining stable receptor interactions, limited ligand mobility, and the most favorable MM/PBSA binding free energy. CHEMBL521996 also demonstrated favorable energetic stabilization despite undergoing a stable positional rearrangement within the binding pocket, whereas CHEMBL1202320 displayed comparatively greater dynamic flexibility. Collectively, these findings identify CHEMBL3236582 as the most promising candidate for future experimental validation and support its potential as a ligand targeting the histamine-associated allosteric pocket of the GABA-A receptor with potential applications in cognitive and neurological disorders.

## Figures and Tables

**Table 1:** Descriptors used in similarity analysis.

DW Descriptor Name	Significance
FragFp similarity	Fragment-dictionary-based binary fingerprint of common substructures; similarity increases with the proportion of fragments shared between two molecules.
PathFp	Hashed binary fingerprint encoding all distinct linear atom–bond paths up to 7 atoms; emphasizes similarity in connectivity and substitution patterns along chains, broadly comparable to classical Daylight-type path fingerprints.
AllFragFp	Hashed 2048-bit fingerprint capturing all substructure fragments with up to 6 connected bonds, including stereochemistry; delivers a more fine-grained, substructure-oriented similarity and supports efficient pre-screening in substructure searches.
SphereFp	Binary circular fingerprint built from concentric “spheres” of atoms around each center; encodes local neighborhood environments and aromaticity, providing an intuitive measure of similarity at the level of rings and scaffolds.
SkelSpheres	Count-based circular fingerprint related to SphereFp that additionally encodes heteroatom-depleted carbon skeletons and stereochemistry; offers fine-grained, chemically intuitive scaffold similarity while tolerating single-atom substitutions.
OrgFunctions	Functional-group-based descriptor that classifies organic and organometallic functional groups and their steric/electronic environment; molecules are considered similar when they present comparable reactive motifs, largely independent of the underlying carbon skeleton.
Flexophore	3D pharmacophore descriptor derived from ensembles of low-energy conformers; encodes spatial arrangements of key interaction features to estimate similarity in potential protein-binding behavior rather than purely 2D graph similarity.

**Table 2:** Highest-ranking molecules from PS set, based on computed similarity scores.

ChEMBL	Structure	GA	GI	S	DCS
CHEMBL1202320	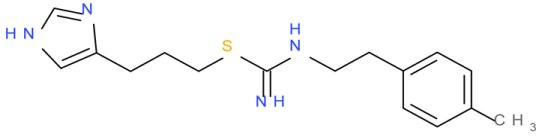	13	3	3.25	6
CHEMBL1202331	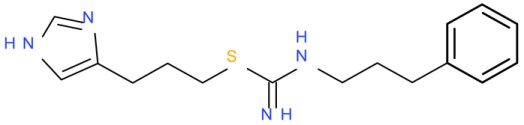	11	0	11	5
CHEMBL1202325	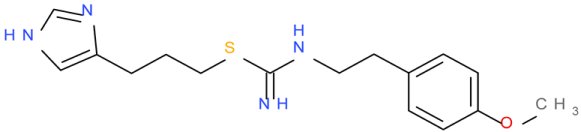	11	1	5.5	6
CHEMBL521996	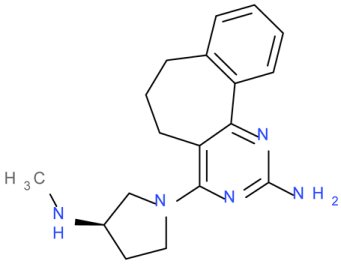	11	1	5.5	6
CHEMBL365257	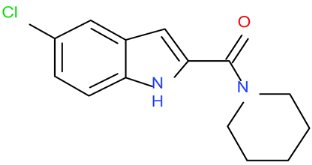	11	1	5.5	6
CHEMBL1923028	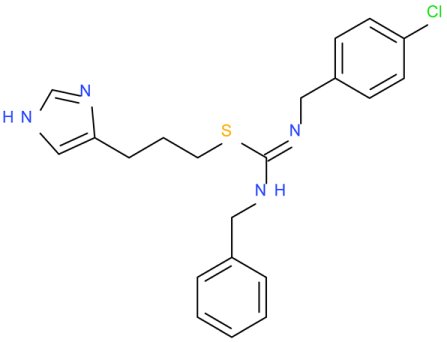	10	0	10	5
CHEMBL197477	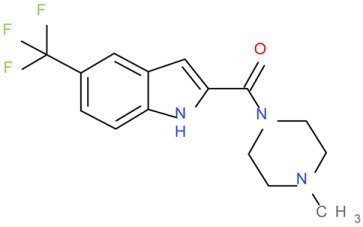	10	2	3.33	5
CHEMBL522673	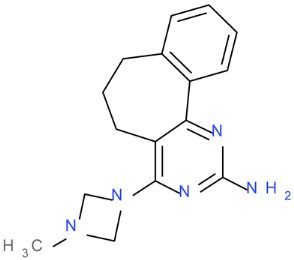	9	1	4.5	5
CHEMBL43934	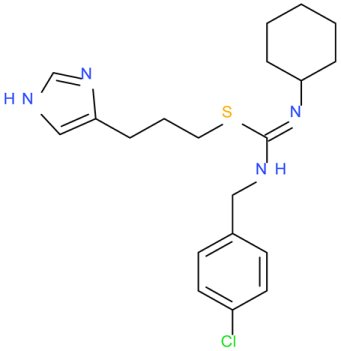	9	1	4.5	4
CHEMBL1923029	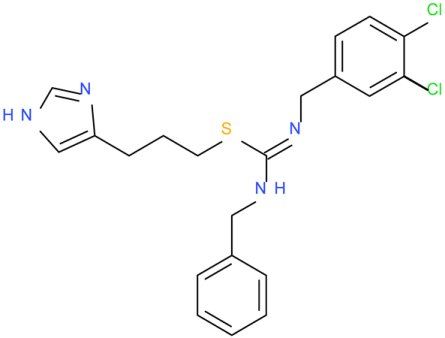	9	1	4.5	4
CHEMBL39705	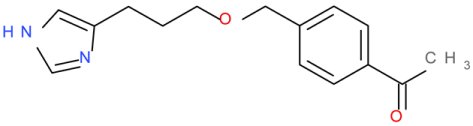	9	1	4.5	4
CHEMBL2047453	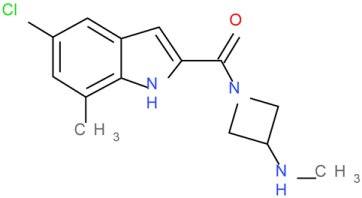	9	1	4.5	3
CHEMBL1923027	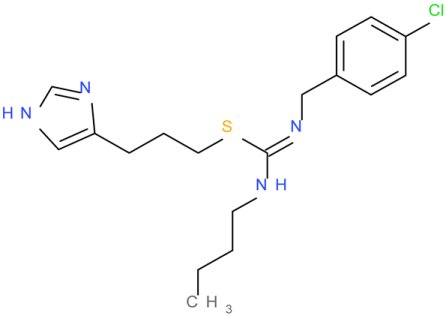	9	1	4.5	2
CHEMBL1923032	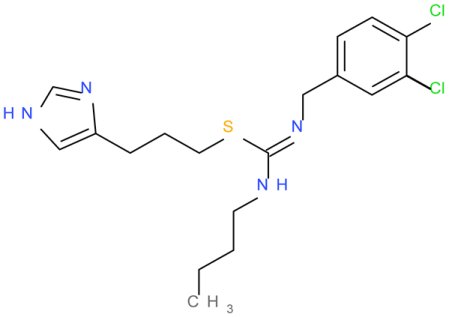	9	2	3	2
CHEMBL3236582	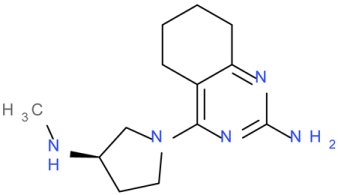	8	0	8	2

GA—global activity score; GI—global inactivity score; S—composite similarity score; DCS—Descriptor Consensus Score.

**Table 3:** Results for the 10 active compounds docked on the homopentameric configuration.

Ligand	ΔG (kcal/mol)	LE	BEI	SEI	LLE	LELP	Class	K_d_ (μM)
(R)-α-Methylhistamine	−5.24	0.582	30.69	7.02	4.17	−0.57	H3/H4 agonist	180
(S)-α-Methylhistamine	−5.50	0.611	32.21	7.37	4.36	−0.54	H3/H4 agonist	51
4-Methylhistamine	−5.14	0.571	30.10	6.89	4.06	−0.51	H4 agonist	32
A-943931	−8.10	0.368	20.10	7.33	3.99	5.29	H4 antagonist	120
A-987306	−7.71	0.321	17.26	7.41	4.31	4.16	H4 antagonist	46
Famotidine	−7.44	0.372	16.16	2.29	6.57	−2.99	H2 antagonist	81
Imetit	−5.78	0.525	24.89	4.08	4.57	−0.63	H3/H4 agonist	51
Immepip	−6.41	0.534	28.43	11.54	4.03	1.25	H3/H4 agonist	69
JNJ-7777120	−7.08	0.373	16.61	33.04	2.04	8.46	H4 antagonist	28
Tiotidine	−7.50	0.375	17.61	2.92	5.93	−1.13	H2 antagonist	33

ΔG—binding energy; LE—ligand efficiency; BEI—binding efficiency index; SEI—surface efficiency index; LLE—lipophilic ligand efficiency; LELP—ligand efficiency-dependent lipophilicity; K_d_—dissociation constant.

**Table 4:** Docking results for the selected 13 hit molecules and their BBB, TOX and DLK scores.

Ligand	LE	ΔG (kcal/mol)	BBB	TOX	DLK
CHEMBL11919	0.627	−5.65	0.868	1	4
CHEMBL13795 (VUF-5297)	0.618	−5.56	0.757	1	4
CHEMBL268229	0.616	−5.54	0.689	1	4
CHEMBL268490	0.528	−4.76	0.560	1	4
CHEMBL2047445	0.474	−8.53	0.797	2	3
CHEMBL2047464	0.451	−8.57	0.964	2	3
CHEMBL197477	0.442	−9.72	0.999	2	4
CHEMBL3236582	0.434	−7.81	0.971	2	3
CHEMBL2047453	0.428	−8.13	0.850	2	3
CHEMBL1202320	0.376	−7.90	0.896	1	3
CHEMBL521996	0.374	−8.61	0.997	2	3
CHEMBL1202331	0.361	−7.57	0.529	2	3
CHEMBL522673	0.314	−6.91	0.998	2	3

LE—ligand efficiency, BBB—blood–brain barrier permeation probability, TOX—number of toxicological alerts, DLK—drug-likeness score.

## Data Availability

The original contributions presented in the study are included in the article, further inquiries can be directed to the corresponding author.

## Abbreviations

The following abbreviations are used in this manuscript:

ADMET	Absorption, distribution, metabolism, excretion, and toxicity
AH	Active-hit set
AUC	Area under the curve
BBB	Blood–brain barrier
BEI	Binding efficiency index
CNS	Central nervous system
DCS	Descriptor Consensus Score
DLK	Drug-likeness score
GA	Global active-similarity score
GI	Global inactive-similarity score
GABA	γ-Aminobutyric acid
GABA-A	γ-Aminobutyric acid type A receptor
LE	Ligand efficiency
LELP	Ligand efficiency-dependent lipophilicity
LLE	Lipophilic ligand efficiency
MD	Molecular dynamics
MM/PBSA	Molecular mechanics/Poisson–Boltzmann surface area
NAM	Negative allosteric modulator
PAM	Positive allosteric modulator
PDB	Protein Data Bank
PME	Particle mesh Ewald
PS	Prioritized Similarity Set
Rg	Radius of gyration
RMSD	Root mean square deviation
ROC	Receiver operating characteristic
SEI	Surface efficiency index
TOX	Toxicity score
